# The dynamic myeloid-enriched microenvironment of glioblastoma: a major challenge to immunotherapy efficacy

**DOI:** 10.3389/fimmu.2026.1755073

**Published:** 2026-02-10

**Authors:** Cindy Lamorlette, Cédric Boura, Sophie Pinel, Danièle Bensoussan, Gianpietro Dotti, Cécile Alanio, Loïc Reppel

**Affiliations:** 1 Université de Lorraine, Centre National de Recherche Scientifique (CNRS), Unité Mixte de Recherche (UMR) 7365 Ingénierie Moléculaire, Cellulaire et Physiopathologie (IMoPA), Nancy, France; 2 Université de Lorraine, Centre National de Recherche Scientifique (CNRS), Unité Mixte de Recherche (UMR)7039 Centre de Recherche en Automatique de Nancy (CRAN), Nancy, France; 3 Centre Hospitalier Régional Universitaire (CHRU) Nancy, Cell Therapy and Tissue Bank Unit, MTInov Bioproduction and Biotherapy Integrator, Nancy, France; 4 Lineberger Comprehensive Cancer Center, University of North Carolina, Chapel Hill, NC, United States; 5 Université Paris Cité, Inserm, Paris Cardiovascular Research Center (PARCC), Paris, France; 6 Department of Neurosurgery, Perelman School of Medicine at the University of Pennsylvania, Philadelphia, PA, United States; 7 Biology Department, Foch Hospital, Suresnes, France

**Keywords:** glioblastoma, immunotherapy, microenvironment, modulating factors, myeloid cells

## Abstract

Glioblastoma (GBM) is the most common and aggressive brain tumor in adults, and current treatments remain poorly efficient. In this context, immunotherapies may represent promising strategies. However, their efficacy is often limited by a strong negative impact of the tumor microenvironment (TME) of glioblastoma. Several factors such as tumor cells mutational profile, previous lines of conventional treatments, or biological factors, have been shown as involved in TME modulation. In this review, our goal is to give an overview of the main modulating factors of the TME of glioblastoma tumors. We will also highlight the importance of developing complex and integrative models to study this microenvironment. At the end, by highlighting critical components of the glioblastoma microenvironment, this review aims to support the development of next-generation, more effective and personalized immunotherapeutic strategies.

## Graphical Abstract

**Figure:**
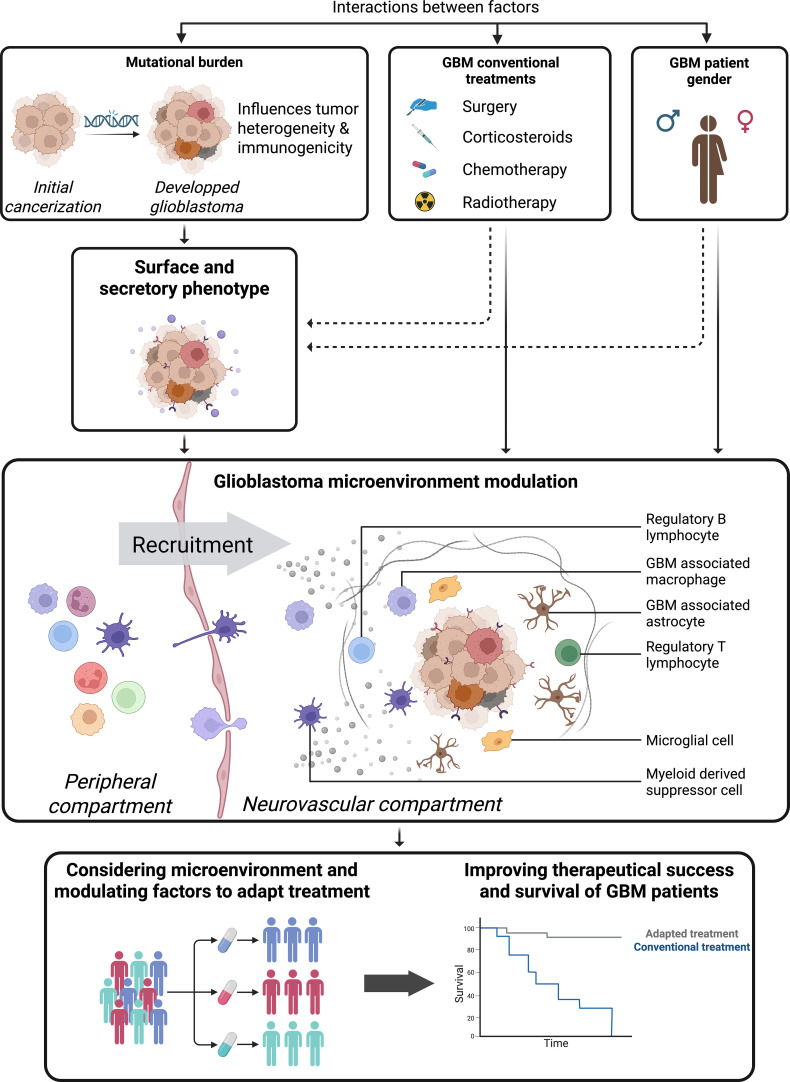
Created in BioRender. Lamorlette, C. (2026) https://BioRender.com/0j9owwq.

## 1 Introduction

Glioblastomas (GBMs) account for 50% of Central Nervous System (CNS) malignancies, making them the most common cancer in this category. Incidence typically ranges between 3 and 5 cases per 100000 person-years (1). In 2025, more than 24000 new cases of nervous system cancers and over 18000 related deaths are expected, with glioblastoma accounting for a significant proportion (2). The latest World Health Organization classification, updated in 2021, defines glioblastomas as tumors without mutations in the genes encoding isocitrate dehydrogenase (IDH) and histone H3. The diagnosis also includes histological criteria such as microvascular proliferation and necrosis. In addition, some IDH-wild type grade II and III diffuse astrocytomas can, in some cases, be reclassified as GBM if they show some molecular alterations (3). The median survival of glioblastoma does not exceed 15 months post-diagnosis, highlighting their aggressiveness and the need to improve patients management (1).

The current standard of care for glioblastoma relies on maximal safe resection, followed by radiotherapy combined with the alkylating agent Temozolomide (TMZ), as established by the Stupp protocol (4). This multimodal regimen temporarily delays tumor progression and modestly improves overall survival, with median outcomes of about 14–18 months. However, nearly all patients experience relapse within the first year after diagnosis (5, 6). Despite progress in surgery and adjuvant therapy, GBM remains incurable due to its highly invasive nature and resistance to conventional treatments (7–9). In recent years, immunotherapy has emerged as a promising avenue to overcome these therapeutic limitations. Several clinical trials have evaluated immunotherapeutic approaches including immune checkpoint inhibitors, dendritic cell vaccines, or adoptive cell transfer in patients with GBM (10, 11). However, most of these strategies have not achieved durable clinical responses, largely because of the profoundly immunosuppressive tumor microenvironment that characterizes GBM (12, 13). In the development of GBM, molecular and morphological changes are induced by inflammation and anarchic vascularization, caused by the disease itself and the treatments administered (5, 14–16). Together, these changes contribute to increase vascular permeability within the blood-brain barrier (BBB), leading to extravasation of peripheral blood cells into the brain parenchyma. Consequently, glioblastoma TME is composed of cell types originating both from the brain or from the bloodstream. These cells are diverted from their physiological role in favor of tumor growth, and thus contribute to the acquisition of treatment resistance and invasive capabilities by glioblastoma cells (17). In this review, we discuss the intrinsic and extrinsic factors influencing the tumor immune microenvironment of GBM, and the therapeutic prospects that would arise from a better understanding of them.

## 2 Glioblastoma immune microenvironment: a myeloid populations enriched tumor environment

### 2.1 GBM microenvironment

The tumor microenvironment of glioblastoma is highly complex and dynamic. Distinct niches result from interactions between tumor cells and diverse non-malignant cells (18–20). These interactions are mediated by soluble factors, extracellular vesicles, and direct cell–cell communication, including gap junctions (21).

Tumor cells and glioma stem cells (GSCs) are central players in modulating the TME. GSCs, characterized by their tumor-initiating potential, heterogeneity, and self-renewal capacity, maintain stemness through markers such as Sox2 and Oct4. Beyond their intrinsic aggressiveness, they actively contribute to immunosuppression through previously mentioned interactions. These mechanisms can promote regulatory T cell expansion *via* TGF-β, induce cytotoxic T cells apoptosis through galectin-3, and drive tumor associated macrophages (TAM) polarization toward an M2-like phenotype by secreting arginase and periostin (22, 23).

The predominant immune component of the GBM TME is the myeloid cells compartment. TAMs represent up to 30–50% of the tumor mass, with approximately 85% originating from bone marrow–derived monocytes (BM-TAMs) and the remaining 15% from resident microglia (MG-TAMs) (15). These cells exhibit marked plasticity in response to local signals and, despite frequent classification as M2-like, now clearly extend beyond the classical M1/M2 dichotomy. Functionally, TAMs support angiogenesis, tumor and GSC proliferation, in addition to local immunosuppression (24).

Myeloid derived suppressor cells (CD3^–^/CD19^–^/CD33^+^/CD11b^+^/HLA-DR^low^)(MDSCs) represent another key immunosuppressive population, detected in the spleen, blood, and tumor tissue of GBM patients (25–28). They are classically subdivided into monocytic (m-MDSC, CD14+), granulocytic/polymorphonuclear (g-MDSC, CD15+), and early-stage (e-MDSC, CD14–/CD15–) subsets. Their relative abundance varies across studies (12, 15, 27, 29, 30). MDSCs exert potent local and systemic immunosuppressive effects through PD-L1, arginase, reactive oxygen species (ROS), prostaglandin E2 (PGE2), and inhibitory cytokines. Altogether, these mechanisms promote tumor progression, treatment resistance, and recurrence (25, 26, 31). Interestingly, recent findings have identified CD14^+^/CD15^+^ double-positive MDSCs in several types of cancer, potentially representing a transitional or hybrid state between the classical monocytic and granulocytic subsets. *Sun* et al. *(2021)* described enrichment of such cells following BTK (Bruton’s tyrosine kinase) inhibition in solid tumors; while *Maneta* et al. *(2022)* reported similar populations in the context of neutrophils mobilization after G-CSF administration (32, 33). Although such populations remain poorly characterized in glioblastoma, these observations highlight the plasticity of the MDSC compartment and suggests potential contributions to immune evasion and therapeutic resistance in GBM.

Other immune populations including N2 tumor-associated neutrophils, tolerogenic dendritic cells, exhausted tumor-infiltrating lymphocytes, and a limited number of regulatory T cells, also contribute to immunosuppression within the GBM TME (21, 34). Together, these immune populations create an environment that supports tumor immune escape and impacts immunotherapy efficacy.

Beyond immune cells, GBM also influence surrounding non-immune brain cells, including glioblastoma-associated astrocytes (GAAs), neurons, oligodendrocytes, endothelial cells, and microglia (20, 35). Particularly abundant at early stages of tumor growth, these populations promote invasion, angiogenesis, and metabolic reprogramming while further impairing anti-tumor immune responses (36).

### 2.2 GBM associated myeloid cells as a continuum of cellular states

Myeloid cells and microglia represent the most abundant populations within the GBM tumor microenvironment (20, 37). Accounting for 30–70% of all cells in glioblastoma, their high prevalence correlates with poor clinical outcomes and reduced patient survival (15, 34). This strong association underscores their central role in tumor progression and highlights the myeloid compartment as a promising therapeutic target for improving the efficacy of anti-GBM strategies.

**Figure 1:**
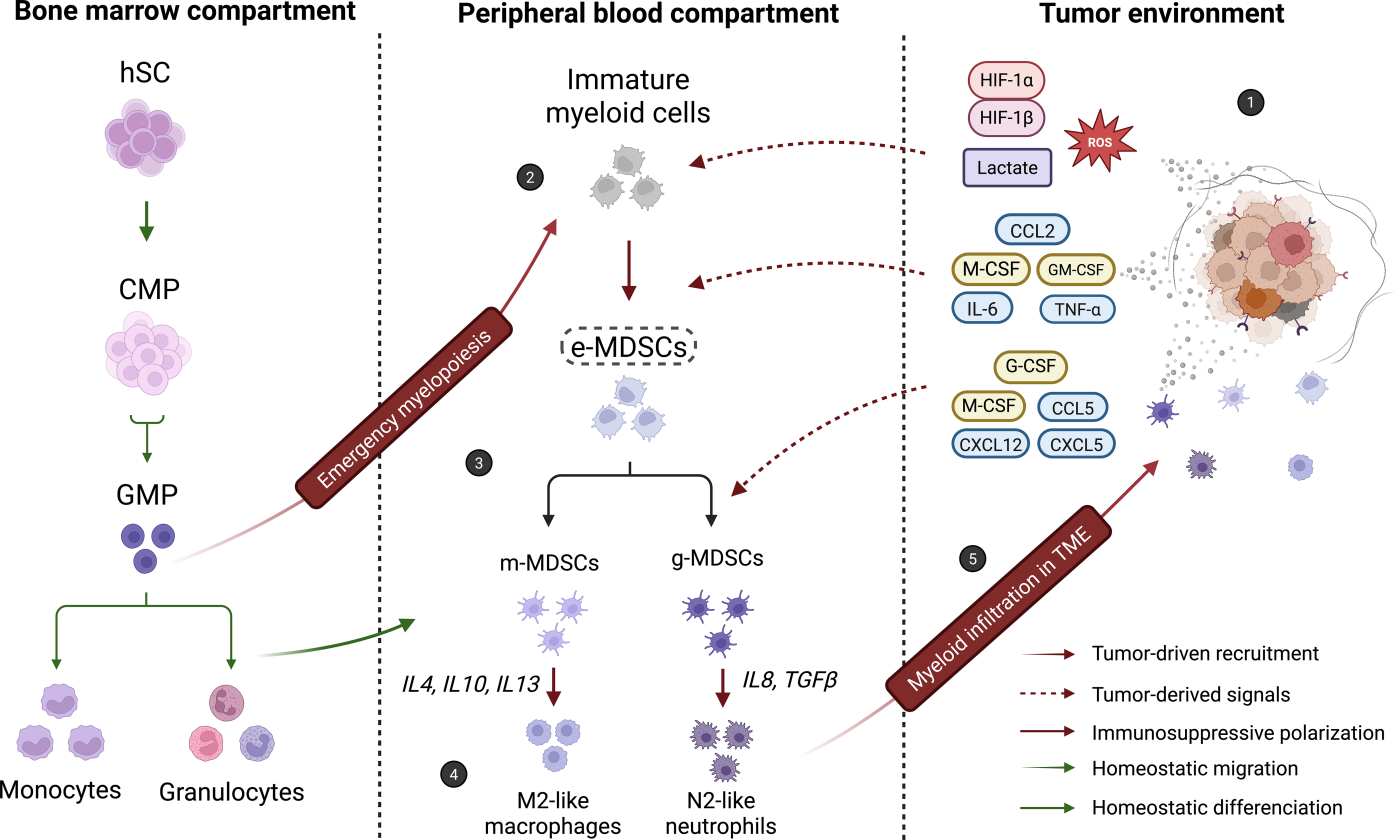
Schematic representation of a proposed myeloid cells plasticity model in GBM. In physiological conditions, hematopoietic stem cells (HSCs) differentiate into common myeloid progenitors (CMPs) and granulocytic–monocytic progenitors (GMPs) within the bone marrow, giving rise to mature monocytes and granulocytes that migrate through the peripheral blood. In tumoral conditions, however, hypoxia and acidosis within the tumor microenvironment (TME) (1) trigger emergency myelopoiesis, promoting the overproduction of myeloid progenitors and the accumulation of immature myeloid cells in the peripheral blood (2) (105). Under the influence of tumor-derived factors, early-stage MDSCs (e-MDSCs) expand and accumulate (3). This transient population has been reported to display monocytic (m-MDSC) or granulocytic (g-MDSC) features under tumor-derived signaling pathways. From these subsets, M2-like macrophage and N2-like neutrophil phenotypes have been described under the influence of M-CSF, IL-4, IL-10, IL-13, IL-8, and TGF-β, respectively (106, 107) (4). Finally, both immature myeloid populations and pro-tumoral myeloid cells are recruited into the TME, where they further polarize and reinforce immunosuppressive networks (5). Importantly, this schematic presents a conceptual model mainly based on preclinical studies. Lineage relationships among myeloid subsets in human GBM remain to be fully established. Created in BioRender. Lamorlette, C. (2026) https://BioRender.com/uipgq82.

Several studies have explored the relationship between myeloid-derived suppressor cells and tumor-associated macrophages. While TAMs have been extensively characterized as key drivers of glioblastoma progression, MDSCs remain comparatively less studied in this context. Accumulating evidence suggests that MDSCs may contribute to tumor progression at earlier stages and could influence the composition and function of myeloid subpopulations, including TAMs and tumor-associated neutrophils (TANs). Based on these observations, it has been proposed that tumor-infiltrating myeloid populations may represent a continuum of phenotypic and functional cell states (**Figure 1**). This continuum model has been described in different solid tumors, and several studies in glioblastoma support similar patterns of myeloid plasticity. Together, GBM-specific data align with broader cancer findings, although the precise lineage relationships remain unclear (38).

MDSCs and TAMs share many properties, the first being their recruitment to the tumor site. Recruitment and maintenance of these cells depend on numerous cytokine and chemokine axes, such as TGF-β, TNF-α; and CCL2/CCR2, CCL5/CCR5, CXCL5/CXCR2, CXCL12/CXCR4. Growth factors involved in their expansion and survival within the TME include G-CSF, M-CSF and GM-CSF. They also share many phenotypic characteristics, notably those related to immunosuppression, angiogenesis and the acquisition of invasiveness by tumor cells (25, 39, 40).

Within this conceptual model, early-stage MDSCs or less differentiated MDSCs have been proposed as potential precursors of more specialized myeloid subsets. Monocytic-like or granulocytic-like MDSCs may in turn acquire TAM- or TAN-like phenotypes depending on their polarization trajectory and local signals (**Figure 1**) (12, 41).

This hypothesis is supported by experimental studies, such as those used by *Kumar* et al. *(2016) (*38). This review is based on multiple *in vitro*, murine, and human research highlighting the ability of myeloid cells to modulate their phenotypes in response to TME signals. It therefore demonstrates the capacity of MDSCs to progressively shift from their role as modulators of immunity to pro-tumoral effectors cells, through signaling pathways involving STAT3 and STAT5 (38).

However, a complex set of signals act synergistically to promote this process. Importantly, hypoxia, defining feature of the GBM, has been shown to enhance MDSCs immunosuppression and may also promote their transition toward macrophage-like states (38). In line with this*, Li* et al. *(2021)* reported accelerated differentiation of monocytic MDSCs into TAMs under hypoxic conditions in experimental models (42). However, most evidence supporting this hypothetical continuum derive from preclinical studies. Besides, the precise relationship between subpopulations and differentiation pathways remains uncomplete due to current limitations in lineage tracing and phenotypic plasticity understanding.

Altogether, these findings suggest MDSCs may constitute an important source of immunosuppressive TAMs and TANs (**Figure 1**). Finally, by defining all myeloid cells subsets as relevant therapeutic targets, this model could help orientating the future research for antitumor strategies development. But while this continuum model provides a strong conceptual framework for myeloid cells plasticity understanding, it remains to be fully validated in human disease.

### 2.3 Hypoxia as a contributing factor for immunosuppression

Oxygen levels in human tissues range from approximately 1% in organs such as the thymus and bone marrow to about 14% in the lungs, compared with 21% in ambient air (43). In tumors, oxygen availability can drop to extremely low levels, reaching ~0.3% in some solid cancers. In glioblastoma, intratumoral oxygen tension has been estimated around 1.25%, making hypoxia a prominent and persistent feature of the GBM tumor microenvironment (44).

Beyond its metabolic consequences, hypoxia acts as a central regulator of immune function within the TME. Stabilization of hypoxia-inducible factors, particularly HIF-1α, leads to broad transcriptional reprogramming in tumor and microenvironmental cells, affecting cellular metabolism, oxidative stress responses, and immune regulation. These changes promote anaerobic glycolysis, lactate accumulation, and extracellular acidosis, while simultaneously depleting glucose, amino acids, and oxygen from the local milieu. Together, these constraints impair the effector functions of antitumor immune cells, notably T cells and NK cells.

Hypoxia-driven signaling also directly impacts immune cell phenotypes. HIF-1α has been shown to regulate the expression of immune checkpoint molecules such as LAG-3, PD-1, and CTLA-4 in T cells, contributing to metabolic dysfunction, exhaustion, and reduced antitumor activity (5, 45). In parallel, hypoxic conditions favor the differentiation and expansion of regulatory T cells through Foxp3 upregulation, reinforcing local immunosuppression (46).

Importantly, hypoxia amplifies the immunosuppressive and pro-tumoral functions of myeloid populations. As discussed above, metabolic reprogramming under hypoxic conditions enhances the suppressive functions of MDSCs and TAMs by increasing the expression of iNOS, arginase, and PD-L1 (38, 42, 45). In addition to its effects on immune cells, hypoxia supports glioma stem cell maintenance, invasiveness, and resistance to therapy, thereby contributing to tumor recurrence and progression (22, 47).

The consequences of hypoxia are not limited to the tumor site. Increased blood–brain barrier permeability facilitates the release of tumor-associated and immunosuppressive cells into the circulation. Populations such as MDSCs can exert systemic immune effects which may be exacerbated by standard treatments, including radiotherapy and chemotherapy (48).

Hypoxia represents a defining component of the glioblastoma microenvironment. Through spatial gradients and metabolic stress, it acts as a key driver of the functional reprogramming described in the GBM TME. While hypoxia plays a central role in tumor immune evasion and therapeutic resistance, it acts in parallel with multiple factors. Together, genetic, biological, and treatment-related factors contribute to the dynamic complexity of glioblastoma pathophysiology.

## 3 TME modulation is influenced by many factors

### 3.1 Mutational burden and microenvironment modulation

Glioblastomas are classically divided into proneural, classical, and mesenchymal molecular subtypes, each associated with distinct immune features. Proneural tumors generally display weaker immune responses, often linked to low HLA-I expression. Besides, classical GBMs show variable immunogenicity depending on the balance between pro-inflammatory and immunosuppressive signals. Finally, the mesenchymal subtype, the most aggressive, is characterized by high expression of cytokines and immune-system interactions molecules. These differences are reflected in the tumor microenvironment, as increasing tumor aggressiveness correlates with higher densities of myeloid infiltrates and immunosuppressive signatures (5).

This association highlights a close link between tumor phenotype and immune modulation, suggesting that higher immunogenicity could promote enrichment of suppressive myeloid populations. However, GBM subtypes do not represent fixed entities but rather form a dynamic continuum, with frequent transitions, especially from proneural to mesenchymal states, and marked intra- and inter-tumoral heterogeneity (14). While this phenomenon complexifies immune profiling and therapeutic targeting, it may also influence immune infiltration. Finally, even though GBMs rarely conform to a strict warm/cold tumor classification, mutational burden and immunogenicity must be considered as antitumor responses modulators.

Comprehensive studies integrating these parameters in GBM remain limited. Nonetheless, *DeCordova* et al. *(2020)* identified DNA methylation–based GBM subtypes associated with distinct immune infiltration patterns. For example, they reported enhanced myeloid recruitment driven by NF1 deficiency. Consistently, *Linxweiler* et al. *(2020)* showed in salivary gland carcinomas that low mutational burden favors T-cell exclusion and myeloid accumulation within the TME. These findings support the idea that the mutational and neoantigenic landscape can shape tumor immune composition (49).

Another example of molecular regulation involves gangliosides, plasma membrane glycolipids participating in cell signaling through interactions with other membrane molecules (50). The expression of certain gangliosides, such as disialogangliosides GD2 (GD2), is frequently dysregulated in GBM (51–53). Most gangliosides exert immunosuppressive effects by inhibiting immune responses, promoting regulatory populations, impairing antigen presentation (50). In addition, some gangliosides were shown to promote MDSCs recruitment and enhance their suppressive activity within the TME (54).

These data suggest that genetic, epigenetic, and metabolic alterations jointly shape the GBM immune microenvironment, especially the myeloid compartment, and represent key levers to overcome GBM-associated immunosuppression.

### 3.2 Modulation of the TME by conventional treatments

As previously mentioned, GBM diagnosis leads to the administration of a standard treatment regimen combining surgical resection with temozolomide (TMZ) chemotherapy and radiotherapy (RT) (5). Corticosteroids such as dexamethasone are also administered to reduce cerebral edema, but these treatments are not without consequences for patients (55).

Several teams have begun to study the impact of these treatments on the modulation of the TME. Dexamethasone exerts a potent anti-inflammatory effect by inhibiting the production of pro-inflammatory cytokines, stabilizing the BBB and decreasing vascular permeability. This action relieves the symptoms experienced by GBM patients, but it may also limit the infiltration of beneficial immune cells and thus impair the antitumor response. Furthermore, dexamethasone also limits effector T-cell infiltration, and promote regulatory immune populations within the TME, raising concerns regarding its prolonged use (55).

Temozolomide is a triazene derivative undergoing rapid chemical conversion after administration. The resulting product is an alkylating agent that induce DNA damage leading to apoptotic death of tumor cells. However, lymphopenia is a common consequence at both low and high doses, resulting in impaired immune competence (56, 57). At lower doses, TMZ has nonetheless been reported to modulate antitumor immunity, although results remain heterogeneous and appear dose-dependent (56).

Radiotherapy has a more localized action, inducing radiation-induced DNA damage. Despite effective tumor cell killing, most recurrences arise within irradiated regions, highlighting the impact of RT on TME dynamics. RT alters structural environment, impacting tissue architecture, extracellular matrix composition, vascular permeability, and hypoxic gradients (39, 58). These changes are associated with microglial activation, recruitment of myeloid cells, and polarization of macrophages toward pro-tumoral M2 phenotypes. These effects are further amplified by hypoxia and ROS and, collectively, favor tumor cell survival, invasion, and immune evasion (58, 59).

More recently, RT has also been shown to induce senescence in GBM cells, including glioma stem cells. The resulting senescence-associated secretory phenotype (SASP) reinforces immunosuppressive and pro-tumoral signaling. By promoting resistance to apoptosis, and supporting the formation of post-treatment survival niches, RT further contribute to tumor recurrence (58–60).

While many studies focus on monotherapies, TMZ and radiotherapy are generally administered in combination, and this strategy has been shown to induce immunosuppressive remodeling of the TME. For example, increased recruitment of m-MDSCs by endothelial cells and elevated circulating MDSCs in lymphopenic patients are reported after chemo-radiotherapy (61, 62). These findings support a synergistic effect of conventional therapies on myeloid-driven immunosuppression.

In conclusion, the GBM microenvironment is shaped not only by intrinsic tumor biology but also by standard therapeutic interventions, adding an additional layer of complexity to GBM pathophysiology.

### 3.3 Patient gender: a promising yet little-explored avenue

Genetic and hormonal differences between the sexes significantly influence immune function. Females generally exhibit stronger innate and adaptive immune responses than males (63). Sex-related disparities have been reported in GBM. The incidence of GBM is about 1.6 times higher in men, whereas women show improved survival after diagnosis. Hormonal factors may contribute to these differences, as estrogen has been associated with neuroprotective and anti-inflammatory effects, whereas higher testosterone levels correlates with poorer prognosis (64). These findings suggest that sex may influence both tumor development and the immune landscape of the TME.

Beyond hormonal effects, sex-associated genetic and epigenetic differences have also been described. Increased MGMT promoter methylation, linked to improved response to chemotherapy, has been reported more frequently in female GBM patients (65), although benefits are observed in both sexes (66). Additional studies have identified sex-specific transcriptional programs related to hypoxia, inflammation, and genomic instability, which are known modulators of immune cells behavior within the TME (67).

Experimental models further support sex-dependent immune regulation. In murine GBM models, sex-specific differences have been observed in myeloid populations, including differential regulation of monocytic and granulocytic MDSCs, with distinct therapeutic responses to MDSC targeting strategies (68). Sex-dependent differences in lymphoid compartments have also been reported in mouse models, where females exhibit enhanced CD8^+^ T-cell responses and improved survival (69). While emerging evidence from patient cohorts suggests similar trends in T-cell dysfunction and exhaustion, these observations remain largely associative in human GBM.

These studies show that sex is an important biological variable associated with immune and myeloid heterogeneity in glioblastoma. However, current evidence, largely derived from preclinical models and retrospective patient analyses, supports an associative rather than predictive role of sex-based immune signatures. Prospective studies will be required to determine their clinical relevance and potential utility in therapeutic stratification.

## 4 Immunotherapies and microenvironment in glioblastoma

### 4.1 Autologous cell therapies and their undeniable efficiency

Numerous studies have investigated the efficacy of immunotherapies (ITs) for the treatment of GBM (20, 48, 70–72). Early approaches mainly focused on immune checkpoint inhibitors (ICIs), including anti-CTLA-4 and anti-PD-1/PD-L1 antibodies. Despite their significant success in several types of cancer, resistance to ICIs frequently emerges (73). In fact, used as monotherapy, ICIs have failed to demonstrate significant clinical benefit in GBM patients (74, 75). The mechanisms underlying these failures remain incompletely understood. *Alban* et al. *(2024)* reported a selective activity of nivolumab on weakly immunogenic tumor clones, associated with TME remodeling, suggesting that immune pressure may favor tumor adaptation and survival (76). These observations highlight complex and underexplored interactions between therapeutic strategies, tumor mutational profiles, and the microenvironment.

Synergistic effects have been reported when ICIs are combined with chemo- and radiotherapy. In the CheckMate-143 trial (Phase I, *NCT02017717*) nivolumab combined with standard treatment was associated with a doubling of median survival in MGMT-methylated GBM patients. RT and chemotherapy may enhance tumor immunogenicity and increase BBB permeability, thereby facilitating ICI efficacy (39, 77). However, increased mutational burden does not consistently translate into improved responses to ICIs, and treatment-induced TME remodeling may also promote pro-tumoral pathways, resulting in heterogeneous clinical outcomes (77). Finally, the characteristics of GBM make this type of tumor a poor candidate for the use of ICIs as monotherapy.

Alternative immunotherapies are therefore being considered, including autologous cell-based therapies (70, 78). These approaches rely on the patient’s own immune cells and include dendritic cell (DC) vaccines, NK cell–based therapies, and T-cell–based strategies (71). DC vaccines, such as DCVax-L (*NCT00045968*), have shown encouraging results, although their highly personalized nature limits large-scale applicability. NK cells represent another attractive option due to their ability to target tumor cells with reduced HLA expression. However, their short lifespan and difficulties in sustained activation remain major challenges (79).

In recent years, T cells have increasingly demonstrated their potential. Tumor-infiltrating lymphocytes (TILs) offer high tumor specificity but are strongly influenced by solid tumor characteristics limiting their isolation and expansion. Transgenic TCR-engineered T cells overcome some of these limitations by targeting tumor neoantigens and can be generated from peripheral blood, with promising results reported in GBM (79–81). Among these strategies, chimeric antigen receptor (CAR) T cells currently represent the most advanced targeted approach. CAR-T cells recognize surface antigens independently of HLA presentation, allowing broader antigen targeting in GBM (82). Although major challenges remain for CAR-T efficacy in solid tumors, multiple GBM-associated antigens targeting shew encouraging outcomes (11, 83, 84).

The efficacy of immunotherapies depends on the patient’s prior antitumor immunity, which is strongly impacted in GBM. The establishment of protumoral TME contributes to the acquisition of treatment resistance. However, the mechanisms linking immune dysfunction, treatment response, and TME remodeling remain poorly defined, underscoring the need for more representative experimental models.

### 4.2 Involvement of TME in therapeutical failures: How to explore it?

As discussed above, MDSCs, TAMs, and microglial cells implement distinct yet interconnected immunosuppressive mechanisms, placing the myeloid compartment at the center of current investigations (85–87). MDSCs, which may act upstream of other suppressive myeloid populations, are of particular interest. However, deciphering these mechanisms remains challenging due to the lack of representative experimental models. We acknowledge TME is a highly dynamic environment shaped by continuous changes influencing GBM progression and therapeutic response (88). And most *in vitro* and *in vivo* studies are carried out in incomplete models, that fail to adequately recapitulate the complexity of the myeloid-enriched TME, limiting the predictive value of immunotherapy efficacy. Consequently, this leads to promising *in vitro* results, followed by failure or recurrences in pre-clinical and clinical conditions (83, 84).

This limitation likely contributes to the lack of clinical benefit previously described for ICIs. In addition to the immunosuppressive MET, ICI efficacy depends on the presence of functional antitumor immune cells, particularly T cells. However, as highlighted earlier, peripheral T-cell depletion and the low lymphoid infiltration frequently observed in GBM represent major obstacles to effective immune checkpoint blockade. Similar constraints apply to CAR-T cell therapies. CAR-T cells must overcome local and systemic immunosuppression that promotes T-cell exhaustion and limits persistence, but also physical barriers. Continuous exposure to suppressive cells and soluble factors ultimately leads to functional exhaustion and progressive loss of antitumor activity, as reported in multiple CAR-T studies (82, 83).

In response to this growing need to understand interactions within the TME, various models have been set up, each recapitulating distinct aspects of the GBM microenvironment. Two-dimensional *in vitro* models, typically based on GBM cell lines or primary tumor cells, are easy to implement but fail to account for TME complexity. Three-dimensional spheroid models better reproduce tumor architecture but remain limited by the difficulty of incorporating and maintaining multiple TME components. Organoids represent one of the most physiologically relevant *in vitro* approaches, preserving key features of patient tumors. However, their reproducibility is constrained by tumor heterogeneity and non-physiological culture conditions. These strengths and limitations are summarized in **Table 1**, which highlights the relevance of each model for studying myeloid–immunotherapy interactions (88).

**Table 1:** Strengths and limitations of experimental models to investigate myeloid-driven immunosuppression in glioblastoma.

Model	Main characteristics	Strengths	Limitations	Relevance for myeloid studies	Suitability for immunotherapy testing
2D GBM cell lines	Established or primary GBM cells cultured in monolayers	Simple, reproducible, cost-effective	No TME, no immune compartment	Low	Low
2D co-culture systems	GBM cells with selected immune/myeloid populations	Controlled interactions, mechanistic studies	Artificial ratios, reduced complexity	Moderate	Moderate
3D spheroids	Tumor cells organized in 3D aggregates	Recapitulate architecture and gradients	Limited immune integration, short-term cultures	Moderate	Moderate
GBM organoids	Patient-derived 3D cultures preserving heterogeneity	High relevance to human tumors	Variable reproducibility, limited immune cells	Moderate	Moderate
Syngeneic mouse models	Murine GBM cells in immunocompetent mice	Intact immune system, TME interactions	Murine immune bias	High	Good
Genetically engineered mouse models (GEMMs)	Spontaneous tumors driven by oncogenic alterations	Tumor–TME co-evolution	Limited human mutational relevance	High	Good
Patient-derived xenografts (PDX)	Human GBM tissue implanted in mice	Patient relevance	Immunodeficient hosts	Moderate	Low
Humanized mouse models	Human immune system reconstituted in mice	Partial human immune context	Incomplete immune reconstitution	Moderate	Moderate
Advanced hybrid models	Organoids + immune cells / engineered niches	Increased complexity	Technical variability	High	Moderate

This table summarizes commonly used *in vitro* and *in vivo* models, highlighting their main characteristics, strengths, and limitations. The relative relevance of each model for investigating myeloid cell biology and for evaluating immunotherapy efficacy is indicated.

*In vivo* models provide additional insight into TME-driven mechanisms. Xenograft models derived from patient samples or tumor cell lines require immunocompromised hosts and are therefore poorly suited for immune studies. Humanized mouse models partially address this issue but fail to fully recapitulate a functional human immune system. Conversely, immunocompetent models allow investigation of immune–tumor interactions, but rely on murine immune systems and do not fully reflect the mutational landscape of human GBM (88). As previously discussed, differences in genetic and phenotypic profiles profoundly affect TME composition and function, further complicating model selection.

No single experimental system fully recapitulates GBM pathophysiology, and each model contributes complementary information. Progressive improvement of these approaches has increased our understanding of TME interactions and facilitated the identification of new therapeutic targets. Evaluating immunotherapies in complex models has highlighted the need for combination strategies that target not only tumor cells but also the immunosuppressive microenvironment. Consistent with this, several combination immunotherapy approaches have already shown promising results *(***Table 2***)*, reinforcing the central role of myeloid cells as therapeutic targets in GBM.

**Table 2:** Completed with results interventional clinical trials reporting the safety and efficacy of combination therapies including at least one immunotherapy for GBM patients (old and new classification).

Clinical trial number	Condition	Date	Combined strategy	Phase	Number of inclusions	Location	Published results
NCT00589875	Malignant glioma	2007-03 2016-08	AdV-tk + Valacyclovir gene therapy + standard radiation therapy	II	52	USA	Demonstrated a favorable tolerability profile and induction of local and systemic immune activation. The use of this strategy as an adjuvant to surgical resection has improved overall and progression-free survival (94).
NCT00643097	Newly diagnosed EGFRvIII+ GBM multiforme	2007-09 2016-11	PEP-3 vaccine + sargramostim (GM-CSF) + TMZ	II	40	USA	Favorable tolerability profile and increased anti-EGFRvIII antibody titers in 85% of patients. Combining this strategy with the standard treatment regimen could offer survival benefits for EGFRvIII+ GBM patients (95).
NCT01081223	Recurrent grade IV gliomas	2010-04 2011-03	Cancer vaccine + immune adjuvant	I - II	12	USA	Completed without conclusion. 100% all-cause mortality and 100% serious and other adverse effects.
NCT02529072	Recurrent brain tumors	2016-01 2019-12	Nivolumab (anti-PD1) + DC vaccines	I	6	USA	Enhanced immune activation with increased tumor-specific T cells. Some patients showed a slowdown in disease progression, but this remains to be confirmed (limited sample) (96).
NCT02968940	IDHmu glioma transformed to GBM	2017-03 2019-08	Avelumab (anti-PDL1) + hypofractionated radiation	II	6	USA	Completed without conclusion. +66% all-cause mortality, +83% serious adverse events and 100% other adverse events.
NCT04225039	Recurrent GBM	2020-06 2025-06	Anti-GITR agonist + PD-1 inhibitor + stereotactic radiosurgery	II	39	USA	Slightly increased toxicity, but still feasible and safe. Immune responses are stimulated and survival is prolonged (75).
NCT04396860	Newly diagnosed MGMT unmethylated glioblastoma	2020-09 2025-03	Ipilimumab (anti-CTLA4) + Nivolumab (anti-PD1) + radiation therapy *VS* usual treatment (Temozolomide and Radiation Therapy)	II - III	159	USA	Higher immune toxicity than TMZ, requiring adjustments in adverse event management. Limited survival benefit compared with TMZ (97).
NCT03426891	Histologically confirmed grade IV malignant glioblastoma or gliosarcoma	2018-03 2025-10	Chemotherapy + radiotherapy + Pembrolizumab (PD-1 inhibitor) + Vorinostat (histone-deacetylase inhibitor)	I	21	USA	Completed without conclusion. Acceptable tolerance without obvious efficacy improvement.

### 4.3 Targeting the GBM TME: MDSCs and immunotherapies

As previously discussed, MDSCs are highly represented in the GBM TME, and are increasingly recognized for their ability to inhibit immune cells activation and function, thereby limiting the efficacy of immunotherapies (86, 87).

Targeting MDSCs therefore represents a promising strategy to improve immunotherapy outcomes, and several approaches have been explored. One option consists in depleting MDSCs using cytotoxic agents. *Kamran* et al. *(2017)* reported that treatment with 5-fluorouracil or gemcitabine selectively reduced MDSC populations in a syngeneic GBM mouse model (85, 86). However, the specificity of these approaches toward MDSC versus other myeloid subsets (e.g., neutrophils or monocytes) remains a point of investigation. Blocking MDSC recruitment or maintenance within the TME has been proposed. For example, through interference with pathways such as GM-CSF or CCL2/CCR2, which promote a reduction of both MDSCs and TAMs accumulation (85, 86). Another strategy aims to inhibit MDSCs immunosuppressive functions using agents targeting Arg-1, inducible nitric oxide synthase (iNOS), or NRF2 signaling pathways (86). Finally, promoting MDSCs differentiation using agents such as all-trans retinoic acid (ATRA) has also been explored, although further data are required to validate this approach (86).

Experimental studies further support the benefit of combining MDSC targeting therapies with other immunotherapies. *Kamran* et al. *(2017)* reported that MDSC depletion or inhibition restored immunostimulatory gene therapy–induced CD8^+^ T-cell responses and significantly prolonged survival in an immunocompetent GBM mouse model (85). More recently, *Zannikou* et al. *(2023)* showed that CAR-T cells engineered to express IL-15 fused to the CAR receptor effectively eliminated MDSCs in both *in vitro* and *in vivo* GBM models. In this setting, IL-15Rα–expressing myeloid cells were also targeted, enhancing CAR-T efficacy and improving survival (89). These promising results justify the growing interest in understanding MDSC-mediated resistance mechanisms and developing strategies to counteract them.

Translational relevance is further supported by clinical data. In a phase I clinical trial, *Peereboom* et al. *(2019; NCT02669173)* reported that daily low-dose capecitabine reduced circulating MDSCs and anti-inflammatory cytokines while increasing cytotoxic T-cell levels in GBM patients. Importantly, patients showing immune restoration exhibited a trend toward improved survival, suggesting that MDSC-targeting strategies could be integrated and effective into combination therapeutic approaches (90).

The rationale for selectively targeting MDSCs is supported by their marked expansion in GBM patients. As MDSCs also contribute substantially to the TAM compartment, their targeting may represent an efficient upstream strategy. However, identifying optimal targets remains challenging, and selective depletion may induce compensatory immunosuppressive mechanisms. This highlights the need for combination strategies targeting both tumor cells and the TME.

## 5 Immunotherapies optimization to improve the management of GBM patients

### 5.1 Combine therapies for the treatment of GBM

Numerous trials are currently investigating combination strategies to improve immunotherapy efficacy in GBM. For example, *Kamran* et al. *(2017)* highlighted the potentiating effect of combining immunotherapies. It finally shew that the use of ICI combined with the immunostimulatory gene therapy TK/Flt3L improved its efficacy (85). Similarly, the use of Nivolumab (anti-PD1) with anti-GD2 CAR-T cells also demonstrated promising results in a mouse model of GBM (53). These results suggest that immune checkpoint blockade, which shows limited efficacy as monotherapy in GBM, may potentiate immunostimulatory approaches when used in combination.

More recently *Martins’ team (2024)* also reported improved CAR-T cell efficacy using CD47 receptor blockade. By promoting phagocytosis of tumor cells by myeloid cells, especially microglia and macrophages, CD47 inhibition acted synergistically with CAR T cell therapy (91). Earlier studies combining anti-CD47 agents with TMZ or radiotherapy had already suggested therapeutic benefit (92). But promoting antitumor myeloid activity, strategies aiming to reduce myeloid immunosuppression have also been explored. Inhibition of CSF-1 receptor signaling, for example, limits interactions between tumor cells and TAMs. When combined with anti-PD-1 therapy, this approach showed beneficial effects, further supporting the relevance of targeting the myeloid compartment to improve immunotherapy efficacy (93).

Overall, combination strategies integrating immune checkpoint inhibitors with radiotherapy, CAR-T cells with TME-targeting agents, or vaccines with immunostimulatory adjuvants appear among the most promising approaches (72).

Numerous clinical trials are currently ongoing, and several have already reported encouraging results consistent with preclinical findings (**Table 1**). While combination therapies generally prolong patient survival, outcomes remain heterogeneous, and some approaches have failed. The specific immunosuppressive characteristics of the GBM microenvironment remain a major obstacle. Combination strategies are promising but must be accompanied by further optimization of immunotherapies (72).

### 5.2 Other approaches to potentialize immunotherapies

**Figure 2:**
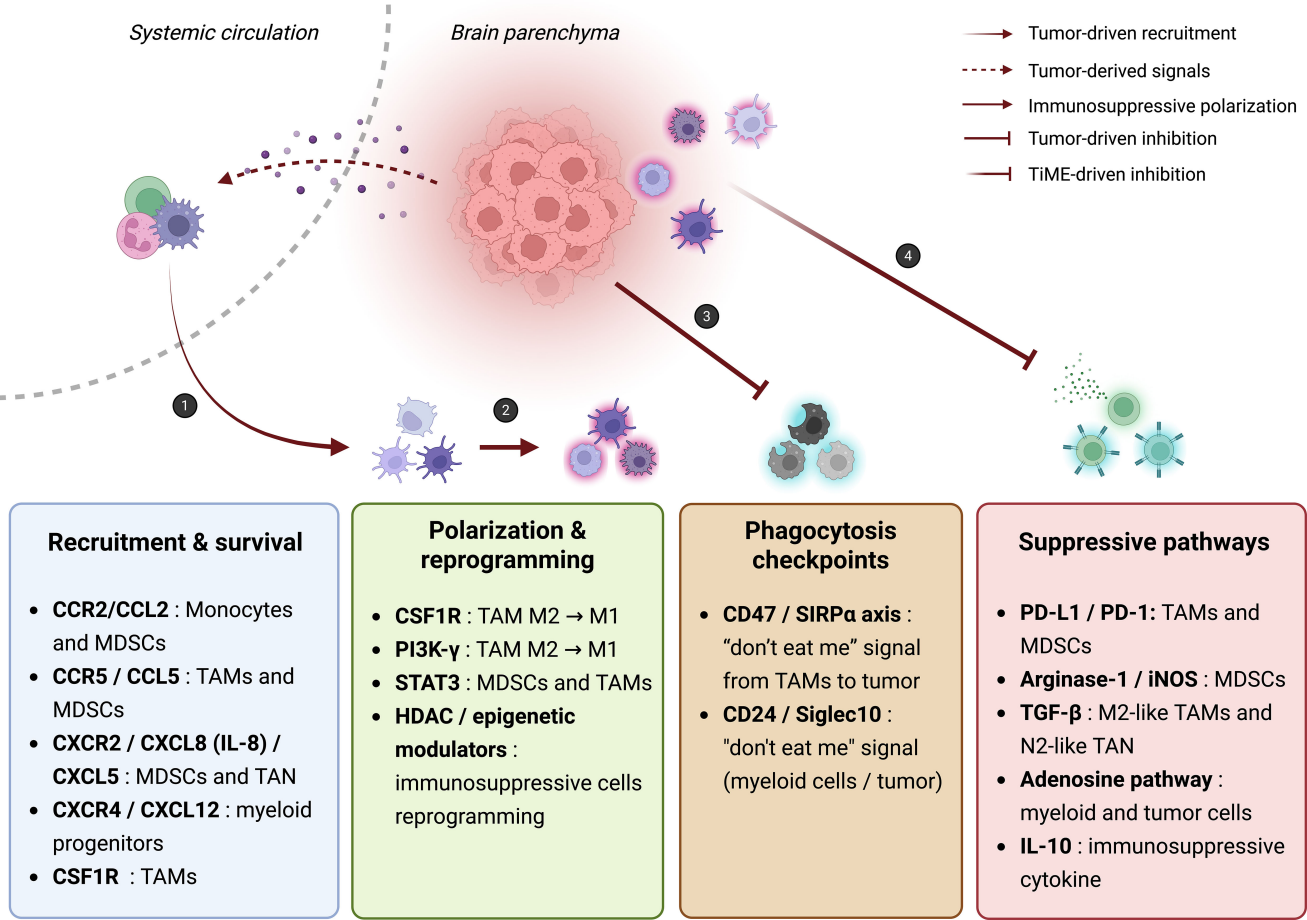
Myeloid-enriched glioblastoma microenvironment and main therapeutic targets. (1) Glioblastoma cells and glioma stem cells remodel the brain parenchyma into a myeloid-enriched, immunosuppressive TME through hypoxia and secretion of CSF1, GM-CSF, IL-6, CCL2, CCL5, CXCL8, and CXCL12, which recruit and sustain peripheral myeloid cells (5, 108). CCR2/CCL2, CCR5/CCL5, CXCR2/CXCL8, CXCR4/CXCL12, and CSF1R pathways mediate their recruitment and survival (109, 110). (2) Once inside the TME, CSF1R, PI3Kγ, and STAT3 signaling polarize macrophages and MDSCs toward immunosuppressive phenotypes, reinforced by HDAC-dependent epigenetic programs (111). (3) Tumor cells evade phagocytosis through CD47/SIRPα and CD24/Siglec-10 “don’t-eat-me” checkpoints that inhibit macrophage and microglial clearance (112, 113). (4) Additional suppressive pathways—including PD-L1/PD-1, Arginase-1/iNOS, TGF-β, and adenosine (CD39/CD73-A2A)—further dampen anti-tumor immunity (10, 114, 115). IL-10 secretion by myeloid cells consolidates immunosuppression (108). These interconnected processes illustrate how recruitment, reprogramming, and suppressive signaling by myeloid cells sustain GBM immune evasion and highlight key therapeutic entry points. Created in BioRender. Lamorlette, C. (2026) https://BioRender.com/jj9joiy.

Optimizing immunotherapy is not limited to therapeutic combinations and encompasses several complementary strategies. Given the strong influence of the tumor microenvironment on treatment response, patient selection based on predictive biomarkers is increasingly recognized as essential (94). The study of the genetic and molecular characteristics of GBMs has already enabled the identification of important markers. These include, for example, MGMT promoter methylation as predictive of TMZ response, or immune infiltration and immunosuppressive signatures conditioning response to immune checkpoint inhibitors. The identification of biomarkers could refine patient stratification and improve the selection of optimal combinations (**Figure 2**), thereby reducing therapeutic failure and unnecessary toxicity.

Improving immunotherapy delivery represents a major area of development. The presence of the BBB limits drug penetration, and direct intratumoral injection or convection-enhanced delivery have been proposed to enhance local efficacy (95, 96). However, given the invasive nature of these approaches, alternative strategies such as sequential administration may represent a more acceptable option (97). Nanoparticle-based delivery systems are also under investigation, although deeper optimization is required (98). Finally, improving delivery methods may enhance therapeutic efficacy while reducing systemic toxicity.

Immunotherapeutic strategies themselves are also undergoing constant innovation. Genetically modified immune cells, especially CAR cells are at the forefront of research. Many teams are increasing their resistance to TME-mediated immunosuppression, though, for example, the addition of cytokines such as IL-15 to the CAR construct. Other optimizations can include engineering CAR cells to resist suppressive signals to improve tumor infiltration, approaches that have shown promising preclinical results (99, 100).

More recently, concepts for manipulating tumor and immune cells metabolism have emerged as an additional avenue to improve immunotherapy efficacy. Tumor metabolic activity contributes to TME remodeling and immune suppression, while immune cells undergo metabolic reprogramming that alters their function. Targeting these metabolic pathways, for instance by reducing tumor acidosis, limiting lactate accumulation, or restoring immune cell metabolism, may enhance antitumor immunity (101). In this context, modulating hypoxia-related pathways, including HIF signaling, could improve CAR-T cell persistence and antitumor activity within the hypoxic GBM microenvironment.

Finally, inhibition of the intrinsic tumor immune evasion mechanisms could be considered. Through reduced expression of HLA molecules, tumor cells can downregulate antigen presentation and escape immune recognition. Restoring HLA-I expression or targeting immune tolerance–inducing molecules such as HLA-G has shown promises in other cancer types and may help restore antitumor immunity in GBM (102–104).

In conclusion, these approaches highlight that no single strategy is likely to overcome GBM-associated immunosuppression. Tumor cells are highly adaptive to their environment, and effective long-term responses will require approaches targeting both tumor cells and the tumor MET cells.

## 6 Conclusion

This review highlights that glioblastoma remains one of the most immunologically challenging solid tumors, largely due to the dynamic and immunosuppressive tumor microenvironment. The abundance and functional diversity of myeloid cells, including TAMs, MDSCs, and microglia, play a central role in shaping this environment, promoting immune escape and contributing to therapeutic resistance. Their important plasticity, driven by tumor and TME derived signals, positions the myeloid compartment as both a major barrier and a relevant target for the development of novel strategies.

Most immunotherapeutic approaches have historically focused on T-cell activation or immune checkpoint inhibition. However, growing evidence suggests that targeting myeloid cells may represent a more effective way to overcome GBM-associated immunoresistance. The identification of transitional myeloid states further supports the concept of a continuum of myeloid polarization and highlights shared suppressive pathways that could be therapeutically exploited. Targeting major suppressive signaling axes in combination with immune checkpoint inhibitors or autologous cell therapies, may therefore provide synergistic benefits by jointly modulating innate and adaptive immune responses.

Finally, future studies should prioritize the use of experimental models that more accurately integrate the spatial and temporal complexity of the GBM microenvironment. Interactions between tumor, stromal, and immune cells, as well as treatment-induced remodeling must be considered in each study. More representative experimental models will be essential to enhance efficacy of therapeutic strategies and improve the clinical management of GBM patients.

## Glossary

Arg-1: Arginase-1
ATRA: Trans-Retinoic Acid
BBB: Blood Brain Barrier
BM-TAM: Bone Marrow derived TAM
BTK: Bruton’s Tyrosine Kinase
CSF: Colony Stimulating Factor
CNS: Central Nervous System
CSF1R: CSF1 Receptor
e-MDSC: early-stage MDSC
Fb: Fibroblast
GAA: GBM Associated Astrocytes
GBM: Glioblastoma
G-CSF: Granulocyte-CSF
GD2: Disialoganglioside GD2
GM-CSF: Granulocyte-Monocyte CSF
g-MDSC: granulocyte-like MDSC
GSC: Glioma Stem Cell
HIF: Hypoxia Induced Factor
IDH: Isocitrate DeHydrogenase
ICI: Immune Checkpoint Inhibitor
iNOS: inducible NO Synthase
IS: Immune System
IT: ImmunoTherapy
M-CSF: Monocyte-CSF
MDSC: Myeloid Derived Suppressor Cell
MGMT: O-6-methylguanine-DNA methyltransferase
m-MDSCs: monocytic-like MDSC
MG-TAM: Microglia derived TAM
NF1: NeuroFibromatosis type 1
NRF2: Nuclear Factor Erythroid 2-related factor
PDL1: Programed-Death Ligand 1
PGE2: ProstaGlandine E2
RT: RadioTherapy
ROS: Reactive Oxygen Species
SASP: Senescence Associated Secretory Phenotype
TAM: Tumor Associated Macrophage
TAN: Tumor Associated Neutrophil
TME: Tumor Microenvironment
TIL: Tumor Infiltrated Lymphocyte
TMZ: Temozolomide
